# A quantitative synthesis of and predictive framework for studying winter warming effects in reptiles

**DOI:** 10.1007/s00442-022-05251-3

**Published:** 2022-09-13

**Authors:** Jeanette B. Moss, Kirsty J. MacLeod

**Affiliations:** 1 Department of Evolution, Ecology, and Behavior, University of Illinois, Champaign, IL 61820 USA; 2 Department of Biology, Lund University, Sölvegatan 37, 223 62 Lund, Sweden; 3 School of Natural Sciences, Bangor University, Bangor, LL57 2UR UK

**Keywords:** Meta-analysis, Winter ecology, Reptile, Climate warming, Brumation

## Abstract

Increases in temperature related to global warming have important implications for organismal fitness. For ectotherms inhabiting temperate regions, ‘winter warming’ is likely to be a key source of the thermal variation experienced in future years. Studies focusing on the active season predict largely positive responses to warming in the reptiles; however, overlooking potentially deleterious consequences of warming during the *inactive* season could lead to biased assessments of climate change vulnerability. Here, we review the overwinter ecology of reptiles, and test specific predictions about the effects of warming winters, by performing a meta-analysis of all studies testing winter warming effects on reptile traits to date. We collated information from observational studies measuring responses to natural variation in temperature in more than one winter season, and experimental studies which manipulated ambient temperature during the winter season. Available evidence supports that most reptiles will advance phenologies with rising winter temperatures, which could positively affect fitness by prolonging the active season although effects of these shifts are poorly understood. Conversely, evidence for shifts in survivorship and body condition in response to warming winters was equivocal, with disruptions to biological rhythms potentially leading to unforeseen fitness ramifications. Our results suggest that the effects of warming winters on reptile species are likely to be important but highlight the need for more data and greater integration of experimental and observational approaches. To improve future understanding, we recap major knowledge gaps in the published literature of winter warming effects in reptiles and outline a framework for future research.

**Supplementary Information:**

The online version contains supplementary material available at 10.1007/s00442-022-05251-3.

## Introduction

Increases in temperature related to global warming have important implications for organismal fitness. Relative to endothermic species that regulate their own body temperature, ectothermic species are physiologically reliant on ambient temperature to direct behavior, growth, and reproduction, and are therefore considered especially vulnerable to projected thermal shifts (Deutsch et al. 2008; Kingsolver et al. 2013; Paaijmans et al. 2013). While a majority of the research examining these effects has focused on temperature changes during annual periods of activity (i.e., when mean temperatures exceed the minimum thresholds required for growth and reproduction; Deutsch et al. 2008; Kingsolver et al. 2013; Paaijmans et al. 2013), thermal conditions can also impart strong effects on ectotherms during periods of non-activity or dormancy, such as during winter. Winter temperatures are rising at a faster rate than summer temperatures (Intergovernmental Panel on Climate Change, 2014), and failure to account for asymmetries in warming patterns is likely to bias insights from climate change research (Speights et al. 2017). ‘Winter warming’, although traditionally studied in high arctic species and cold-adapted mammals, is predicted to also have important (and thus far, underestimated) fitness implications for temperate-zone species (Williams et al. 2015; Johansson et al. 2020), and may be an important source of vulnerability for ectotherms (reviewed in Marshall et al. 2020).

One group of ectotherms for which climate change research has been particularly ‘active-season-biased’ is the non-avian reptiles. Indeed, existing frameworks for assessing vulnerability to temperature change in reptiles focus almost exclusively on activity budgets as a metric for fitness, which by definition can only be measured in the active season (Huey et al. 2009; Kearney 2013). Sinervo et al. (2010) projected devastating global extinction rates across lizard species due to thermally restricted activity during reproductive months, a result echoed by later applications of this framework (Böhm et al. 2016; Pontes-da-Silva et al. 2018; Diele-Viegas et al. 2020). Conversely, by the same metrics cold-climate reptiles could *benefit* from warmer breeding seasons due to release from cold stress and extended activity periods (Chamaillé-Jammes et al. 2006; Clarke and Zani 2012; Caldwell et al. 2015; Cabezas-Cartes et al. 2019; Chukwuka et al. 2021; Muñoz et al. 2021). While rising thermal minima are increasingly recognized as a fitness-relevant dimension of reptile thermal ecology (e.g., warming nights; Clarke and Zani 2012; Moore et al. 2020; Chukwuka et al. 2021; Rutschmann et al. 2021), the effects of warming during the winter period are rarely factored into these projections (but see Zani et al. 2012; Bestion et al. 2015).

Neglecting the effects of warming winters risks biasing predictions about how overall warming trends are likely to impact reptile species. We provide the first quantitative synthesis of work on winter warming effects in this group. We begin by evaluating the literature on reptile winter ecology to identify probable mechanisms through which warming could influence phenology, physiology, and fitness. We then synthesise the existing data on this topic using meta-analysis to investigate whether reptilian responses to increases in winter temperature correspond to our biologically informed predictions. Finally, we outline a framework for future studies on this topic, including highlighting understudied areas. We hope that this discussion will stimulate more research on winter ecology and winter warming effects, particularly in the reptiles.

## Reptile winter ecology and predictions under winter warming

### Strategies for overwintering

For reptiles in temperate regions, the arrival of winter ushers in cooler temperatures, reduced food availability, and fewer thermoregulatory opportunities. Most reptiles are capital breeders, relying on stored energy to fuel reproduction in the spring (Bonnet et al. 1998; Shine 2005). Thus, the optimal overwintering strategy should minimize cold risk and maximize the conservation of, and opportunities to replenish, energetic reserves (Storey 2006; Huey et al. 2021).

A common strategy at mid-latitudes is brumation, a metabolic adaptation for ensuring survival in seasonally cold environments (Hoekstra et al. 2020). Brumation may be achieved passively (i.e., metabolic rate declines with temperature), or actively by entering a true dormant state known as metabolic depression in which metabolic rates drop to just above thresholds required to prevent tissue damage (Patterson and Davies 1978; Hailey and Loveridge 1997). Metabolic depression or downregulation is not limited to extremely cold winters; Brazilian tegus (*Tupinambis merianae*) in relatively warm temperate habitats show a reduction in oxygen consumption during winter months to 20–30% resting rate (de Souza 2004).

Many reptiles seek out solitary or communal hibernacula in winter to minimise exposure to sub-zero temperatures (White and Lasiewski 1971; Ultsch 1989; Rabosky et al. 2012). These retreats may be shallow (i.e., 10–30 cm crevices visible from the surface) or deep (i.e., > 1 m), depending on risk of cold injury (Gregory 1982; Huey et al. 2021). Where contact with ambient ice is unavoidable, physiological adaptations are required for freeze tolerance or freeze avoidance. For example, hatchling painted turtles (*Chrysemys picta*), which overwinter in shallow nests frequently penetrated by frost, show a remarkable capacity for supercooling (Costanzo and Lee 2013) that is facilitated at least in part by freeze-responsive gene expression (Storey 2006). While supercooling capacities vary by population, a lack of predictable correlation with winter severity in species that use deep thermal refugia suggests that behavioral avoidance may shield many taxa from selection on physiological tolerance (Michels-Boyce and Zani 2015).

Over-wintering strategies vary geographically and taxonomically: for example, where some populations [e.g., *Sceloporus graciosus* in central California (Jameson 1973) and *Sceloporus occidentalis* in southern Washington (Tsuji 1988)] are entirely winter-dormant, adapting both their physiology (e.g., reduced metabolism) and behavior (e.g., fasting) to conserve energy, others [e.g., *Sceloporus jarrovii* in Arizona (Ruby 1977) and *Sceloporus occidentalis* in southern California (Tsuji 1988)] are continuously or periodically winter-active, resisting energetic slumps brought on by cooler temperatures through opportunistic basking and metabolic compensation. While the physiological benefits of this latter strategy (e.g., feeding, growth, repair, or progression of embryonic development where offspring are retained in nests/in oviducts overwinter) are assumed to override the metabolic costs, these dynamics likely vary by species and remain an open area of research (Huey et al. 2021).

### Sensitivity to thermal cues

While many organisms rely on photoperiodic cues to signal the changing seasons, reptiles that overwinter underground may receive little to no exposure to these cues. Instead, ambient temperature serves as the principal orchestrator of the onset and termination of dormancy in most temperate reptiles (reviewed in Van Dyke 2014). Just as periods of low temperature can induce gradual entry into dormant states regardless of season (Patterson and Davies 1978; Toledo et al. 2008), increases in air, soil, or hibernaculum temperature trigger emergence from extended hibernation (Grobman 1990; Blouin-Demers et al. 2000; DeGregorio et al. 2017). As warming soils are more perceptible closer to the surface, reptiles in warm localities tend to overwinter at shallow depths, presumably to optimize opportunities for midwinter activity (Huey et al. 2021). In colder localities, conversely, reptiles must retreat deep underground, out of range of surface temperature cues, to avoid risk of freezing (Huey et al. 2021). In these cases, it is possible that *critically low* temperatures generate the thermal gradients within hibernacula that initiate migration to the surface (Lutterschmidt et al. 2006).

Temperature also regulates physiological and behavioural states during brumation. For example, cold exposure primes fat body cycles for seasonal fasting (Derickson 1976) and turtle brains for winter anoxia (Couturier et al. 2019) and is also the trigger for metabolic depression. Sensitivity to these cues may be seasonal or year-round: for example, *Uta stansburiana* show the same metabolic response to extreme low temperatures in summer as in winter (Halpern and Lowe 1968). Finally, environmental temperature plays a critical role in regulating reproductive cycles. In dissociated breeders (i.e., where gonadal recrudescence occurs either in the fall or winter leading up to spring mating, Van Dyke 2014), a prolonged cold period prior to the onset of warm temperatures is a prerequisite to recrudescence and associated reproductive behaviors (Marion 1982; Gavaud 1991; Lutterschmidt 2012), with photoperiod having little to no effect (Aldridge 1975, 1979). Even in species that do not rely on a cold period to trigger recrudescence, gradual warming in late winter and early spring is still the primary cue stimulating reproduction (Tinkle and Irwin 1965; Licht et al. 1969). Evidence from laboratory manipulation experiments implicates the action of environmentally sensitive hormones in entraining biological rhythms to overwinter thermal conditions. For example, in snakes, melatonin transduces temperature cues to the brain’s reproductive axis during hibernation (Lutterschmidt and Mason 2009), providing a mechanism for the thermal regulation of seasonal reproductive cycles.

### Key predictions under winter warming

To predict how warming winters are likely to affect temperate reptiles over the short- to long-term, it is not only important to draw direct links between overwinter temperature and survivorship and/or reproductive success, but also to identify biological processes that are likely to be affected by temperature and incorporate these into a more holistic understanding of fitness. A first major prediction, given the extent to which reptiles rely on thermal cues to trigger the onset and termination of dormancy and the initiation of spring reproduction, is that increasingly warm winters will lead to widespread shifts in phenology. Phenological advancements in response to climate change have been documented across plant and animal species (Parmesan 2007; Cohen et al. 2018) and regional and taxon-specific assessments suggest that reptiles are no exception (Urban et al. 2014; Prodon et al. 2020). Shorter winters and longer growing seasons are expected to have positive impacts on temperate reptiles by extending periods available for activity/reproduction (Adolph and Porter 1993; Sperry et al. 2010), although early emergence could also confer deleterious effects such as greater exposure to late-season frosts (Turner and Maclean 2022).

A second major prediction based on the direct links between temperature and resting metabolic rate in reptiles (Gillooly et al. 2001) is that high-temperature dormancy will hasten energy depletion. For example, hatchling painted turtles (*Chrysemys picta*) catabolize more body-derived fuel sources when overwintering at mild (10 °C) or warm (15 °C) temperatures than at cold temperatures (4 °C)—a sign that they have exhausted carbohydrate and lipid stores (Costanzo et al. 2004; Muir et al. 2013). Increased energy use during periods of dormancy not only reduces body condition upon emergence (Costanzo et al. 2004; Brischoux et al. 2016; Muir et al. 2013) and contributes to increased overwinter mortality (Zani 2008; Zani et al. 2012), but also has the potential to limit energy-demanding activities in the spring, such as migration (Tucker et al. 1998), gametogenesis (Derickson 1976), and breeding (MacLeod et al. 2018).

Finally, changes in overwinter temperature could impart hidden fitness costs by disrupting biological rhythms (i.e., sleep vs wake, non-breeding vs breeding) during dormancy. Short-term increases in winter temperature can stimulate midwinter emergence from retreats (Sperry et al. 2010; Nordberg and Cobb 2016, 2017; Huey et al. 2021). However, because temperatures generally remain below the optima for efficient locomotion and digestion (Ruby 1977; Huey and Kingsolver 1989; Besson and Cree 2011), even successful midwinter hunts are likely to end in a net energy deficit (Sperry and Weatherhead 2012; Nordberg and Cobb 2017) and increase susceptibility to predators (Wilson and Cooke 2004; Sperry et al. 2010; Nordberg and Cobb 2016). Warming winters could also have temporally dissociated effects on reproduction by disrupting physiological and neuroendocrinological mechanisms. For example, male garter snakes overwintered at 10 °C present lower plasma androgens and fewer and smaller gonadotropin-releasing hormone (GnRH) cells in their brains after 16 weeks than males overwintered for the same duration at 4 °C (Lutterschmidt et al. 2022), providing a likely explanation for the observed disruptions to courtship behaviors following a mild winter (Lutterschmidt and Mason 2009).

## Data synthesis—meta-analysis methods and results

### Literature search

To test the predictions outlined above, we compiled existing data from the literature on winter warming effects in May 2020 and April 2022 by searching the ISI Web of Science database (all years inclusive) for studies containing the following key words, singly or in combination: winter, temperature, hiberna*, *overwinter*, winter warming, dorman*, brumat*, *reptile, lizard, snake, turtle*. We also made use of the references cited by Williams et al. (2015) in their review of winter warming effects on terrestrial organisms. We conducted an additional search in August 2021 to widen our dataset to include crocodilians, tuatara and amphisbaenids by including the following terms in combinations as above: crocodil*, amphisbaen*, tuatara. A full list of search term combinations is available in Supplementary Material.

We applied the following criteria for eligibility: (1) studies measured traits in response to experimentally manipulated, or natural variation in, *overwinter* temperatures (where winter boundaries were defined by authors according to regional and species-specific criteria); (2) observational/long-term studies measured responses to natural variation in temperature in more than one winter season; (3) experimental studies manipulated ambient temperature, with a control group for comparison, during the species’ regular winter season. This did not include experimental temperature manipulations deemed ecologically irrelevant (e.g., overwinter treatments were designed to simulate normal summer, not winter temperatures). Using the web application Rayyan (Ouzzani et al. 2016), we removed duplicates and screened abstracts for indications that studies fulfilled these criteria. This search yielded an initial set of 67 studies, which were then evaluated to determine eligibility (see Supplementary Material S1 for full PRISMA statement, sensu Moher et al. 2009). In total, we identified 34 eligible studies.

Study information including sample sizes and information on traits measured was extracted from all studies (details below). Where parameter values were not reported in the text, they were extracted from figures using the R package *metaDigitise* (Pick et al. 2018), WebPlotDigitizer (Rohatgi, 2019), or by contacting authors directly. We collected data on traits categorised as follows: Phenology (pertaining to timing of life history events, such as emergence); Body Condition and Performance (e.g., mass, energy content); Fitness (e.g., survival and reproduction); and Biological Rhythms (hormone titres, metabolic rate) (see Supplementary Table S2 for full list of traits including categorisation).

### Statistical analysis

To account for differences in protocols, parameters measured, and types of calculable effect sizes, experimental (*n* = 12 studies) and observational (*n* = 22 studies) datasets were analysed separately. In both cases, effect sizes were calculated using the *escalc* function in the package *metafor* (Viechtbauer 2010). Effect size signs were adjusted systematically to ensure alignment of biological interpretation in terms of “positive” vs “negative” effects (see Supplementary Table S2 for full list of adjusted signs). For example, an increase in overwinter mortality represents a positive effect size with “negative” effects, and as such the effect size sign was changed to negative. Advances in phenology (e.g., earlier nesting dates) were designated as positive and vice versa. Where there was ambiguity about the assumed benefit of an increase or decrease in a trait value, effect size sign was left unchanged.

In the experimental dataset, we used Standardized Mean Difference as the effect size of interest. For studies that compared more than one treatment group to a shared control group, control sample sizes were corrected (by dividing by the number of treatments) to control for psuedoreplication. In the correlational dataset, we used Fisher’s *r*-to-*z* corrected correlation coefficients. Effect sizes and their corresponding sampling variances were fit to separate multi-level random effects models using *metafor*. In both models, the random term structure accounted for study ID, as well as an observation-level random term to control for overdispersion and account for within-study effect size variance additional to sampling error. To control for phylogenetic effects, models also included relatedness matrices derived from phylogenetic trees specific to each dataset constructed using a synthetic super-tree from the Open Tree of Life database (Hinchliff et al. 2015), accessed and pruned through the R package *rotl* (version 3.0.10, Michonneau et al. 2016). We repeated the same models including trait category as a moderator to determine whether overall effects were significantly driven by changes in a particular type of trait. For the observational dataset, we collapsed the “Biological Rhythms” and “Body Condition and Performance” categories into a single category—“Physiology”—due to low sample sizes. We repeated the same models again this time including taxonomic group as a moderator to test for taxon-specific patterns (tuatara were excluded due to low numbers, *N* = 2 effect sizes). Random term structure in moderator models was as above.

We additionally performed Egger’s test for funnel asymmetry (with square root-transformed inverse *N* as a moderator) for both datasets/models. Neither revealed any significant bias (experimental dataset: *F*_1,98_ = 2.19, *P* = 0.14; observational dataset: *F*_1,61_ = − 0.49, *P* = 0.69; Supp Fig. S3). As is commonly the case in ecological data (Noble et al. 2017), many studies across both datasets contributed multiple effect sizes. To better account for non-independence of data points from the same study, we present model coefficients and confidence intervals derived from robust variance estimation throughout (Hedges et al. 2010).

## Results

**Fig. 1:**
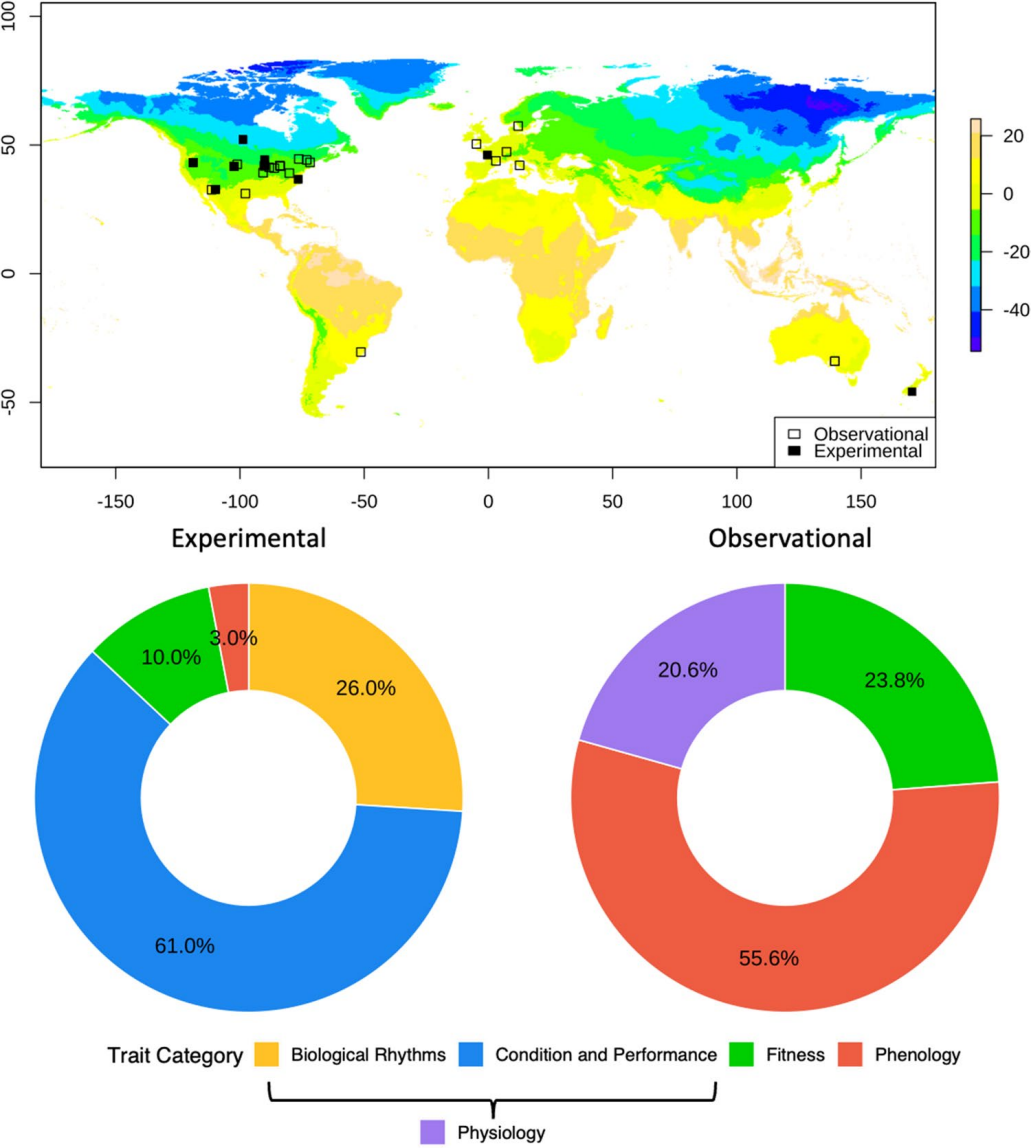
Study sites overlaid on projections of the minimum temperature of the coldest month of the year (in degrees Celsius), estimated by WorldClim and depicted as a colorized raster. Pie charts depict categorical trait representation across experimental studies and observational studies of effects of winter temperature on reptiles

The 34 eligible studies of winter warming effects in reptiles included in our meta-analysis (Supplementary Table 7) hailed from a narrow range of temperate latitudes (34° to 57° N or S). According to WorldClim (Fick and Hijmans 2017), the coldest winter temperatures recorded at these sites between 1970 and 2000 ranged from − 26 to 10.1 °C (Fig. 1). In addition to latitudinal similarities, the majority of studies were geographically clustered: 24 of the 34 were conducted in North America, with an additional 6 in Europe. Only four studies were represented from the whole of temperate regions of Asia, South America, Australia, and Africa. In line with this geographic bias, we also observed considerable taxonomic clustering of studies. Fourteen of the 34 studies focused on North American turtles, whereas studies of lizards (*N* = 9) and snakes (*N* = 11) were more cosmopolitan.

### (***i) Experimental dataset***

Our final experimental dataset comprising 100 individual traits (i.e., effect sizes) from 12 independent studies spanned a limited taxonomic breadth, with data available from 9 species: *Chrysemys picta* (*N* = 4 studies)*, Thamnophis sirtalis* (*N* = 3)*, Uta stansburiana* (*N* = 2), *Vipera aspis* (*N* = 1)*, Sceloporus jarrovii* (*N* = 1), *Sphenodon punctatus* (*N* = 1), *Hoplodactylus maculatus* (*N* = 1), *Naultinus gemmeus* (*N* = 1), and *Oligosoma maccanni* (*N* = 1). The majority (87%) of effects examined related broadly to physiology (i.e., sorted into categories of condition and performance or biological rhythms), including general body condition, energy use, and hormone levels during and immediately following hibernation (Fig. 1a).

**Fig. 2:**
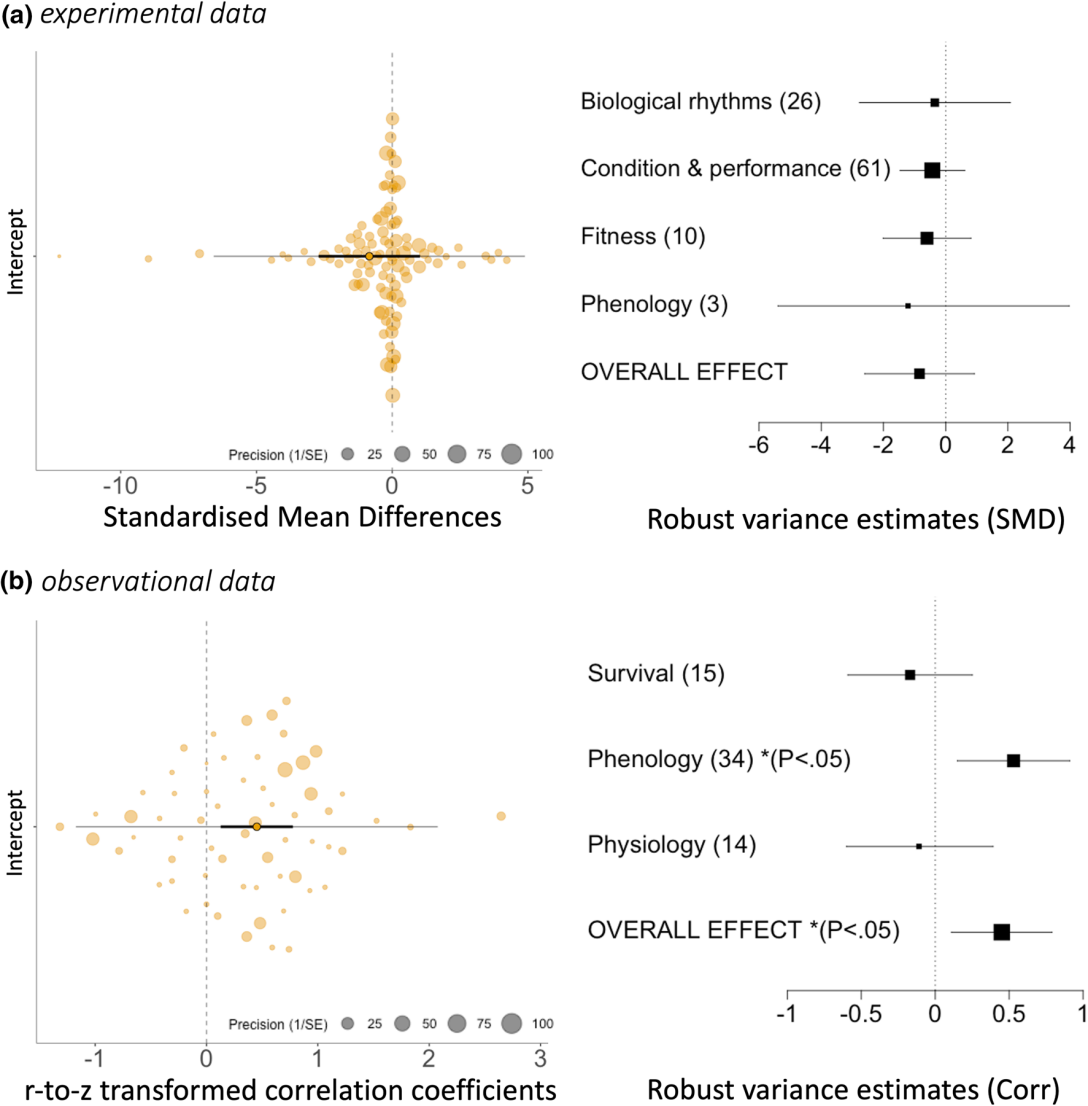
Results from meta-analytical models showing overall patterns of effects sizes in **a** experimental studies (*N* = 12 studies, 100 effect sizes) and **b** obesrvational studies (*N *= 22 studies, 63 effect sizes). Orchard plots (*left panels*) depict overall patterns, with point size corresponding to effect size precision, and meta-analytic means ± CI 95% are depicted as an overlaid *dark horizontal bar*. Overall model heterogeneity is also shown. Forest plots (*right panels*) depict effect sizes grouped by trait category, with coefficients derived from robut variance estimatation presented for each (95% confidence intervals, with estimate depicted by *filled squares*, with *square* size representing estimate precision [1/SE])

There was a non-significant trend for warm winter treatments to have a negative effect on reptile traits (meta-analytical estimate with robust variance estimation − 0.84 ± 0.80, 95% CI − 2.60, 0.92*; P* = 0.32), and this was not driven disproportionately by any trait category (*F*_3,8_ = 0.46*, P* = 0.72; Table 1a; Fig. 2a). Taxonomic group had no effect on overall patterns (*F*_2,9_ = 0.75*, P* = 0.50). Heterogeneity was high (*I*^2^ total = 97.76%), with the majority explained by between-study variance (*I*^2^ study = 78.28%). Heterogeneity attributable to species was low (*I*^2^ species = 0.12%).

**Table 1:** Meta-analytical model tests, coefficients, and confidence intervals derived from robust variance estimation (cluster = study) for (a) experimental data (*N* = 91 outcomes, clusters = 12, mean outcomes/cluster = 7.6); and (b) observational data (*N* = 60 outcomes, clusters = 20, mean outcomes/cluster = 3)

	est	s.e	*F*	*T*	*P*	ci.lb	ci.ub
a. Experimental data							
(i) Overall effect	− 0.84	0.80		− 1.05	0.32	− 2.60	0.92
(ii) Effect of trait category			0.13 (d.f. 3,8)		0.72		
Biological rhythms	− 0.35	1.05		− 0.33	0.75	− 2.77	2.08
Condition/performance	− 0.43	0.45		− 0.95	0.37	− 1.46	0.61
Fitness	− 0.60	0.61		0.98	0.35	− 2.01	0.81
Phenology	− 1.21	1.81		− 0.67	0.52	− 5.39	2.97
b. Observational data							
(i) Overall effect	**0.45**	**0.16**		**2.78**	**0.01**	**0.11**	**0.79**
(ii) Effect of trait category			0.71 (d.f. 2,19)		0.50		
Phenology	**0.53**	**0.18**		**2.92**	**0.01**	**0.15**	**0.91**
Physiology	− 0.11	0.24		− 0.45	0.66	− 0.60	0.39
Fitness	− 0.17	0.20		− 0.68	0.50	− 0.59	0.25

*F* statistics represent the statistical significance of the moderator (trait category) in the basic model (no RVE, ANOVA test). Statistically significant effects (confidence intervals do not overlap zero) are shown in bold

### (***ii) Observational dataset***

Our final observational dataset comprised 63 individual traits (i.e., effect sizes) from 22 independent studies. The taxonomic breadth of these studies was broader than for the experimental dataset (*N* = 26 species) but heavily skewed, with studies of turtles contributing over half of all effect sizes (*N* = 34), and one species alone (*Chrysemys picta*) contributing 17. The majority (53.9%) of traits measured in response to winter temperature were phenological (20 from turtles, 8 from snakes, and 6 from lizards), with survival being the second-best represented trait category (*N* = 15 traits; 11 from turtles, 2 from snakes, and 2 from lizards; Fig. 1b).

While correlations extracted from the observational dataset spanned the full range of possible correlation coefficients (range of correlation values = − 0.866–0.999), the overall trend indicated by our best-fit model was of a significant *positive* fitness effect associated with warmer winter seasons (Table 1b; Fig. 2b; mean r-to-z corrected correlation coefficient = 0.34). Although there were no significant differences between trait categories in the effects of winter warming (*F*_2,19_ = 0.71, *P* = 0.50), the strongest positive effects according to robust variance estimation were in phenological traits (Table 1b). Taxonomic group had no effect on overall patterns (*F*_2,19_ = 0.19*, P* = 0.83). Heterogeneity was also high in this dataset (*I*^2^ total = 99. 85%), with the majority accounted for by between-study variance (*I*^2^ study = 68.81%) and negligible heterogeneity attributable to species (*I*^2^ species < 0.001%).

## Discussion

Non-avian reptiles have featured prominently in studies of biological responses to climate change, with rising thermal maxima and, increasingly, thermal minima implicated in a range of fitness responses (e.g., Sinervo et al. 2010; Bestion et al. 2015; Muñoz et al. 2021). Here, we synthesize our current state of knowledge of *winter* warming effects in reptiles, which have thus far received far less attention. Using a meta-analytical framework, we show that overall effects on reptile traits are generally strong despite overall low availability of data and persistent biases with respect to sampling location and taxonomic group. Below, we outline key emerging trends based on our quantitative synthesis and explore major drivers through a qualitative lens, taking into account taxonomic and geographic biases and differences in study design. We then return to our key predictions to identify pervasive knowledge gaps and develop a framework that builds upon existing empirical knowledge to guide future research on winter warming effects in reptiles.

### (i) Winter warming effects on reptile traits: insights from meta-analyses

Several general patterns emerge from our meta-analysis of existing studies of winter warming in reptiles. First, European and North American taxa (e.g., turtles, especially) are overrepresented relative to other temperate regions. Second, different study designs (i.e., experimental versus observational) support opposing conclusions about the overall effects of winter temperature on reptiles—a not uncommon phenomenon (Wolkovich et al. 2012; Yuan et al. 2017).

#### Reptile phenologies are advancing with the warming of winters

As predicted, warmer winter temperatures were associated with the advancement of phenological traits (i.e., emergence and breeding occur earlier following warmer winters; Fig. 2b). While we have interpreted these effects as “positive”—consistent with the expectation that advancing springs will extend the suitable period for reproduction and offspring growth (Adolph and Porter 1993; Sperry et al. 2010; Clarke and Zani 2012)—the true fitness consequences of changes in phenology likely depend on population-specific environmental factors (Urban et al. 2014; Prodon et al. 2017). In many regions, mild winters are followed by late cold spells, which can expose individuals close to the surface to tissue damage (Benard 2015; Turner and Maclean 2022). Reptiles that specialize on invertebrate prey could also face trophic mismatch, as insects are highly sensitive to short-term climatic shifts and have been shown to advance phenologies at faster rates than insectivorous predators (Vafidis et al. 2019 and references therein). Finally, premature spring emergence could carry reproductive costs. For instance, male lizards that emerge before they reach maximal sperm production engage in many infertile copulations and lose paternity to later emerging males (Olsson and Madsen 1996).

#### Physiological and behavioral disruptions under warming winters

Though environmental temperature is the primary cue by which temperate reptiles coordinate their energy use and reproductive cycles, evidence for disruptive or sublethal effects of high winter temperatures on physiological traits (body condition and biological rhythms) was not statistically significant (Fig. 2), with as many “positive” outcomes reported at warmer winter temperatures [e.g., enhanced basking, foraging, digestive, and/or nutrient assimilation efficiency (Ruby 1977; Besson and Cree, 2011)] as “negative” (e.g., depletion of nutrient stores and acceleration of mass loss) (Ruby 1977; Costanzo 1989; Willette et al. 2005; Zani et al. 2012; Muir et al. 2013; Spencer and Janzen 2014; Brischoux et al. 2016). Strong positive effects appeared to arise more frequently in observational studies, possibly because these are more likely to capture cross-seasonal effects during the growing season that could offset energetic costs (Moore et al. 2020) and risks of mortality incurred overwinter (Zani 2008; Clarke and Zani 2012; Bestion et al. 2015).

In addition to variation between studies, reported effects of winter warming varied within studies dependent on trait, sex, and age. For example, Spencer and Janzen (2014) found inverse effects of winter temperature on rates of mass loss in male versus female hatchling painted turtles. Other studies reported significant alterations to hormonal and behavioral rhythms caused by warm hibernation temperatures (Lutterschmidt and Mason 2009; Brischoux et al. 2016; Lutterschmidt et al. 2022) with unclear fitness repurcussions. For example, neuroendocrine responses to elevated hibernation temperatures appear to delay the peak of male courtship behavior in red-sided garter snakes but do not dampen the performance or body condition of courting males (Lutterschmidt and Mason, 2009). Only two studies in our dataset examined winter warming effects at more than one life-stage, but one of these found opposing effects on adult vs. juvenile survival (Altwegg et al. 2005), again underscoring the point that effects of warming are likely to be complex and multidimensional. Follow-up studies linking fine-grain physiological effects to lifetime whole-organism fitness are needed to improve interpretability of these effects in the future.

#### Winter temperature differentially affects overwinter survival across reptiles

While thermal disruptions to physiology or behavior may be expected to confer indirect effects on survival and reproduction, we found equivocal effects of overwinter temperature on survival across both datasets. Severe *low* temperatures appear to be more limiting for reptile populations at high latitude range boundaries in North America than relatively warm temperatures arising over the same study periods (Packard 1997; Nagle et al. 2000; Kissner and Weatherhead 2005; Baker et al. 2010; but see Zani 2008; Zani et al. 2012), consistent with expectations that ‘winterkill’ is an important source of reptile mortality (Sperry et al. 2010). Despite the clear relevance of these effects for evaluating population vulnerability, we identified only four studies in which overwinter temperatures were experimentaly manipulated to track reptile survivorship (Ruby 1977; Costanzo 1989; Zani 2008; Zani et al. 2012). Only a single study quantified variation in reproductive output in association with winter temperature (finding no difference in the fecundity among gravid *Sceloporus jarrovii* maintained under cold versus warm overwinter temperature regimes, Ruby 1977). Hence, in addition to further study across a wider geographic and taxonomic range, more explicit hypothesis-testing is needed to understand possible population- and species-level variation in sensitivity of fitness-relevant traits to winter warming.

### (ii) Framework for future research

While our meta-analyses expose some intriguing trends, unsurprisingly the consequences of winter warming for reptiles appear complex and multidimensional, and exploration of these nuances remains limited by the number and breadth of relevant studies currently available. Empiricists must, therefore, expand research efforts taxonomically and geographically—work that should become increasingly feasible as wearable technologies become more available and cost-effective (e.g., tracking devices, biosensors; Wilmers et al. 2015). Another important challenge facing current and future researchers in the field is the need to integrate experimental and observational approaches with tests of paired predictions. For example, while longitudinal studies have revealed considerable within- and between-species variation in the magnitude and direction of phenological responses to winter warming (Janzen et al. 2018), persistent gaps exist in our mechanistic understanding of this variation (reviewed in Chmura et al. 2019). Conversely, physiological responses to winter warming have been well studied in experimental contexts, but are poorly documented in natural populations. To enhance this understanding, controlled experiments should ideally be paired with ground-truthing studies in natural populations. Particularly useful would be more holistic studies that investigate effects broadly across trait categories (i.e. not just physiological *or* phenological traits), allowing clearer interpretation of, for example, how changes in physiology or phenology influence body condition or reproductive success.

In addition to biases and methodological inconsistencies, there are key questions surrounding the season-specific effects of temperature in reptiles that are still lacking in basic research. To improve our tools of prediction, experimental work should continue to hone the use of proximate physiological metrics (e.g., hormone profiles, telomere dynamics) to evaluate short- and long-term fitness consequences of winter warming. Reptiles (particularly lizards) are increasingly being used as animal models in general stress studies (Belliure and Clobert 2004; French et al. 2008; MacLeod et al. 2018) and combining hormonal manipulations with manipulations of environmental variables could reveal additive and/or contextual effects that would otherwise be overlooked (Sheriff et al. 2017) such as how overwinter temperatures influence subsequent response to breeding season stressors (MacLeod et al. 2018). There is also a need for more studies investigating how temperature-induced changes in proximate metrics at various points during and after winter hibernation translate into performance to elucidate what constitutes a meaningful deviation in terms of fitness.

Finally, both observational and experimental studies of winter warming in reptiles should integrate carry-over effects to more explicitly link different categories of fitness for which measurement may be temporally decoupled (for example, the long-term effects of physiological costs incurred during dormancy on reproductive performance). An increasing number of studies in reptiles suggest that earlier onset of activity and longer growing seasons, as are anticipated under broadscale spring advancement, could provide a buffer against suboptimal winter conditions, for example by reducing gestational periods (Moore et al. 2020) or increasing energetic stores (Zani 2008; Clarke and Zani 2012; Bestion et al. 2015). Conversely, few studies have investigated how costs incurred during mild winters may carry over to affect energy-demanding tasks (e.g., reproduction, maturation, migration) in the subsequent growing season. Clearly, more long-term studies are needed to ascertain how variables affecting fitness, including winter warming, interact across seasons.

## Supplementary Information

Below is the link to the electronic supplementary material. 

Supplementary file1 (DOCX 6465 KB)

## Data Availability

All data are available at 10.5281/zenodo.7071121.

## Code availability

All code is available at 10.5281/zenodo.7071121.
