# Supplementary material for: A quantitative synthesis of and predictive framework for studying winter warming effects in reptiles
Source: Oecologia. 2022 Sep 13;200(1-2):259–71. doi: 10.1007/s00442-022-05251-3 (PMC9547783; doi:10.1007/s00442-022-05251-3)
Supplement: Supplementary file 1 — Supplementary file1 (DOCX 6465 KB) [file 442_2022_5251_MOESM1_ESM.docx]

**Supplementary Material**

S1. PRISMA [Preferred Reporting Items for Systematic Reviews and Meta-Analyses] statement, *sensu* (Moher et al. 2009).


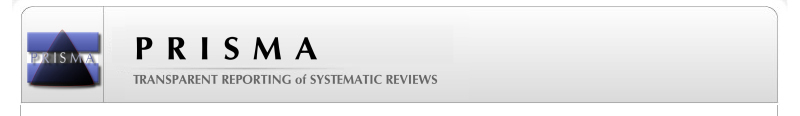
**PRISMA Flow Diagram**

Records identified through database searching
(n = 2421 from WoS )

Full-text articles excluded, with reasons
(n = 29)

Studies included in quantitative synthesis (meta-analysis)
(n = 34)

Studies included in qualitative synthesis
(n = 34)

Full-text articles assessed for eligibility
(n = 67)

Records excluded
(n = 2002)

Records screened
(n = 2069)

Records after duplicates removed
(n =2257)

Additional records identified through other sources
(n = 21)

## Identification

## Eligibility

## Included

## Screening

Search term combinations used: (May 2020/Aug 2021/Apr 2022)

- reptile + winter _ temperature
- reptile + dorman* + temperature
- reptile + overwinter + temperature
- reptile/lizard/snake/turtle + overwinter
- reptile/lizard/snake/turtle + winter warming
- reptile/lizard/snake/turtle + hibernat* + temperature
- reptile/lizard/snake/turtle + dorman* + temperature
- reptile/lizard/snake/turtle + winter + temperature
- crocodil*/amphisbaen*/tuatara + hibernat* + temperature
- crocodil*/amphisbaen*/tuatara + dorman* + temperature
- crocodil*/amphisbaen*/tuatara + winter + temperature
- crocodil*/amphisbaen*/tuatara + winter warming
- reptile/lizard/snake/turtle/crocodil*/amphisbaen*/tuatara +brumat* + temperature

S2. Table of trait categories, and specific traits measured within each. Effect size direction reflects where effect size signs were changed to represent biological reality, i.e., an effect size indicating an increase in a trait was multiplied by -1 where an increase is biologically “negative.” For example, an increase in metabolic rate represents a reduction of energetic reserves. We also interpreted delays in phenology (i.e., longer wait times to an event, such as emergence) as biologically “negative.” These traits were subject to an inverse of sign.

| Trait category | Traits measured | Effect size direction |
| --- | --- | --- |
| Physiology | Body size (e.g., carapace length) | 1 |
|  | Body mass | 1 |
|  | Plasma hormone levels, including change in levels (T/DHT/E2/CORT) | 1 |
|  | Melatonin levels | 1 |
|  | Basking (cloacal) temperature | 1 |
|  | Metabolic rate measures (e.g., rate of O2 exchange) | -1 |
|  | Residual yolk consumption | -1 |
|  | Total energy (e.g., kcal/g) | 1 |
|  | Organic content (including % lipid, % water etc) | 1 |
|  | Liver lipid/glycogen/dry mass | 1 |
|  | Organ mass | 1 |
|  | Non-basking (core body) temperature | 1 |
|  | Levels of inactivity | -1 |
|  | Levels of activity | 1 |
|  | Telomere length | 1 |
| Phenology | Total days in hibernacula | -1 |
|  | Emergence date | -1 |
|  | Oviposition date | -1 |
|  | Incubation duration | 1 |
|  | First emergence/basking (i.e. traits related to onset of annual activity) | -1 |
|  | Emergence synchrony | 1 |
|  | Pairing date | -1 |
|  | Nesting date | -1 |
| Survival | Winterkill (%nests with dead hatch/%mortality) | -1 |
|  | Proportion hatchling survival | 1 |
|  | Adult/juvenile survival probability | 1 |
| Reproduction | Clutch weight | 1 |
|  | Litter size | 1 |
| Behaviour | Nest site selection metrics (e.g. canopy, radiation) | 1 |
|  | Nest depth | -1 |

S3. Funnel plot asymmetry. We performed Egger’s test for funnel asymmetry (with square root-transformed inverse N as a moderator) for both datasets/models – neither revealed any significant bias (experimental dataset: F_1,98_ = 2.19, p = 0.14, Fig S3a; observational dataset: F_1,61_ = 0.16, p = 0.69; Fig S3b).


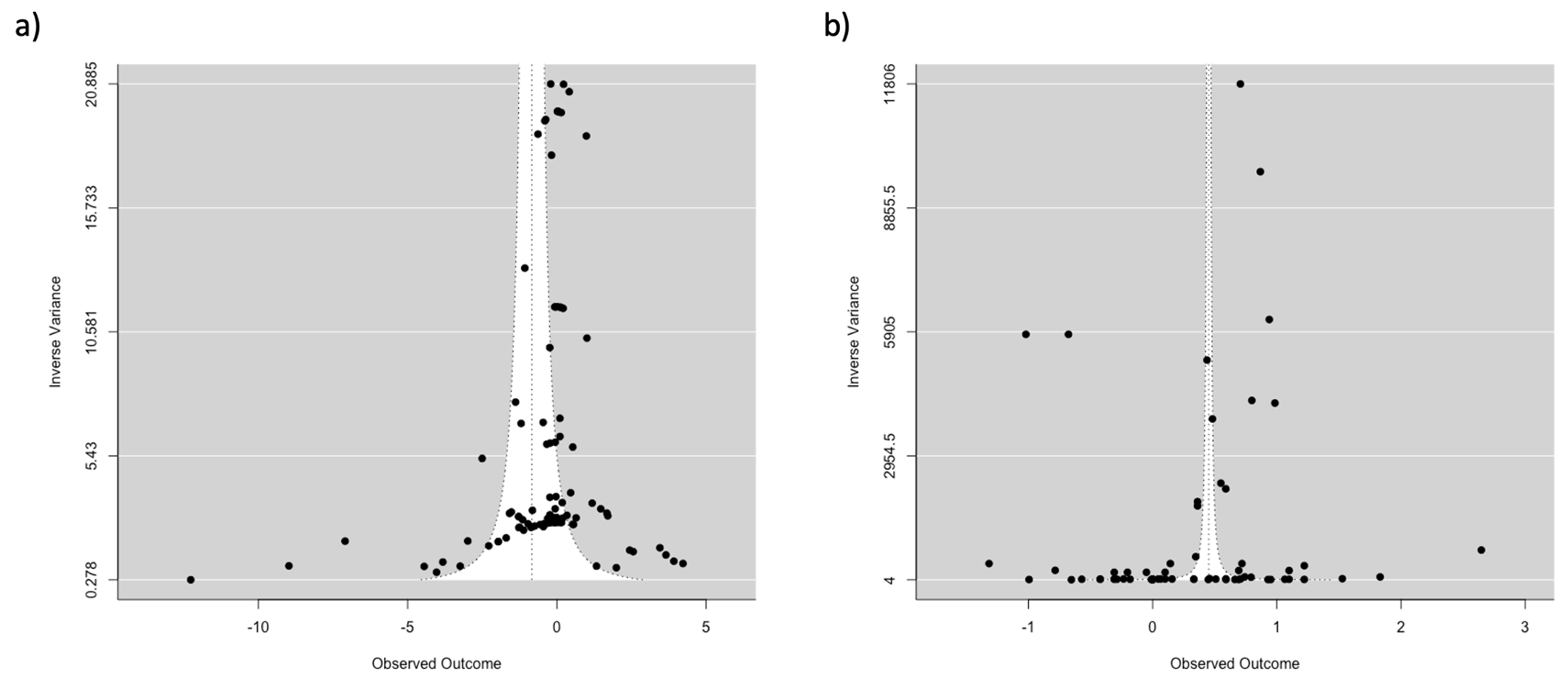


S4. Summary of experimental studies of winter warming effects in reptiles used in meta-analysis.

|  |  |  | Effect sizes | | | | |
| --- | --- | --- | --- | --- | --- | --- | --- |
| Dataset | Trait category | Category represented in N studies | Total effect sizes | Turtles | Lizards | Snakes | Tuatara |
| **a) experimental data** | **Biological rhythms** | **5** | **26** | **6** | **0** | **20** | **0** |
|  | Activity/metabolism | 2 | 6 | 6 | 0 | 0 | 0 |
|  | Endocrine traits | 3 | 20 | 0 | 0 | 20 | 0 |
|  | **Condition/performance** | **9** | **61** | **17** | **30** | **12** | **2** |
|  | Mass measures | 6 | 23 | 9 | 12 | 4 | 0 |
|  | Energy content | 2 | 8 | 6 | 2 | 0 | 0 |
|  | Body composition | 1 | 8 | 0 | 8 | 0 | 0 |
|  | Yolk measures | 2 | 4 | 4 | 0 | 0 | 0 |
|  | Food handling | 1 | 10 | 0 | 8 | 0 | 2 |
|  | Courtship behaviour | 1 | 8 | 0 | 0 | 8 | 0 |
|  | **Fitness** | **4** | **10** | **0** | **9** | **1** | **0** |
|  | Survival | 4 | 6 | 0 | 5 | 1 | 0 |
|  | Reproduction | 1 | 4 | 0 | 4 | 0 | 0 |
|  | **Phenology** | **2** | **3** | **3** | **0** | **0** | **0** |
|  |  |  | **100** | **26** | **39** | **33** | **2** |
|  |  |  |  |  |  |  |  |
| **b) observational data** | **Physiology** | **4** | **11** | **3** | **3** | **7** | **0** |
|  | Activity/metabolism | 2 | 4 | 0 | 2 | 4 | 0 |
|  | Hormone levels | 2 | 3 | 0 | 0 | 3 | 0 |
|  | Telomeres | 1 | 1 | 0 | 1 | 0 | 0 |
|  | Mass measures | 1 | 3 | 3 | 0 | 0 | 0 |
|  | **Phenological** | **11** | **34** | **20** | **6** | **9** | **0** |
|  | Hibernation duration | 2 | 2 | 1 | 0 | 1 | 0 |
|  | Emergence | 5 | 28 | 16 | 5 | 8 | 0 |
|  | Date of First repro | 4 | 4 | 3 | 1 | 0 | 0 |
|  | **Survival** | **7** | **15** | **11** | **2** | **2** | **0** |
|  | Adult | 2 | 2 | 0 | 1 | 1 | 0 |
|  | Neonatal/juvenile | 7 | 13 | 11 | 1 | 1 | 0 |
|  |  |  | **63** | **34** | **11** | **18** | **0** |

S5. Forest plot depicting all effect sizes in experimental dataset (estimate [95%CI])


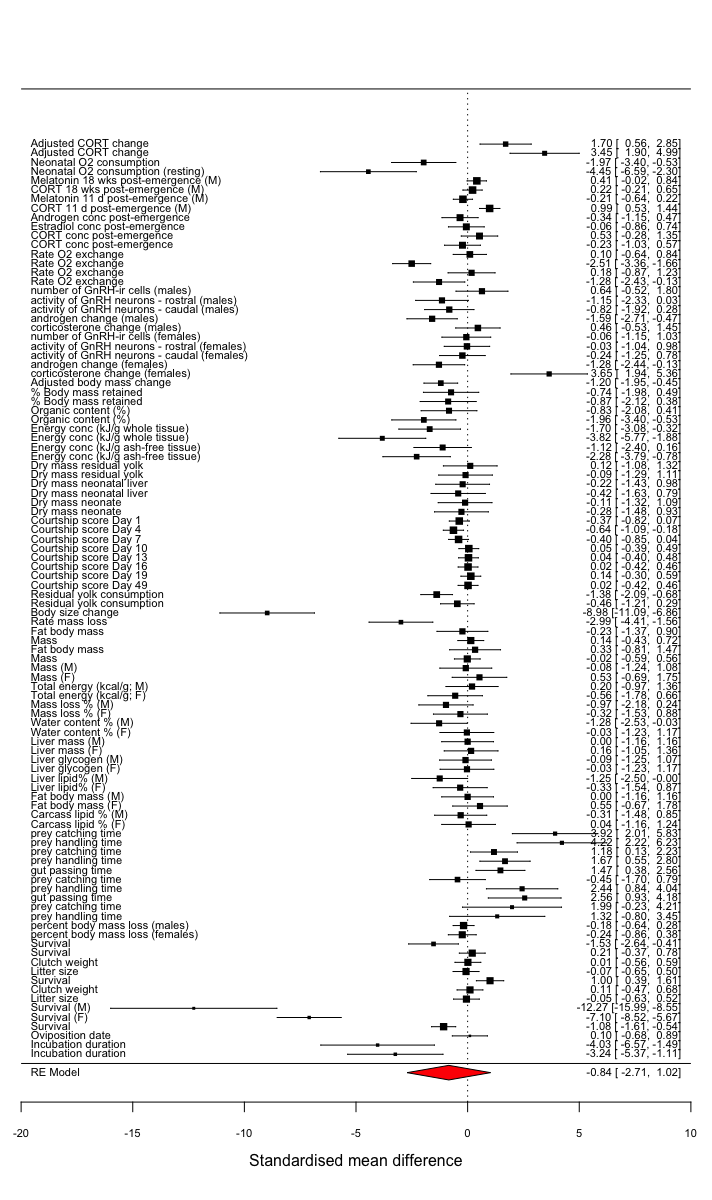


S6. Forest plot depicting all effect sizes in observational dataset (estimate [95%CI])


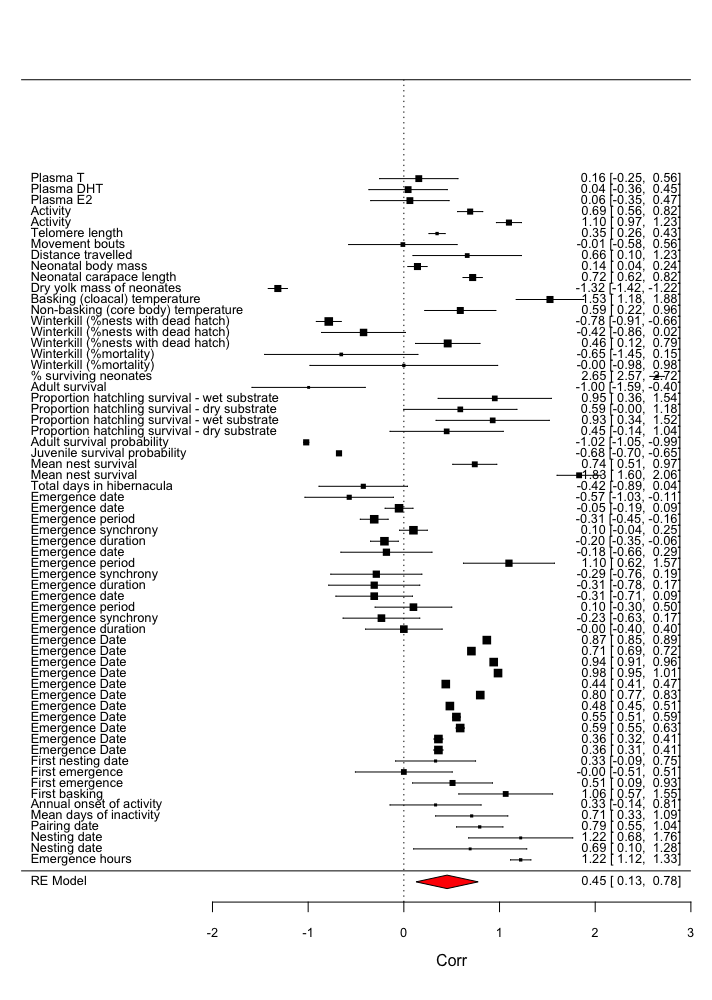


S7. Bibliography of all studies included in the meta-analysis.

a) Experimental dataset

Besson, Anne Amélie, and Alison Cree. ‘A Cold-Adapted Reptile Becomes a More Effective Thermoregulator in a Thermally Challenging Environment’. *Oecologia* 163, no. 3 (July 2010): 571–81. <https://doi.org/10.1007/s00442-010-1571-y>.

Brischoux, François, Andréaz Dupoué, Olivier Lourdais, and Frédéric Angelier. ‘Effects of Mild Wintering Conditions on Body Mass and Corticosterone Levels in a Temperate Reptile, the Aspic Viper (*Vipera Aspis*)’. *Comparative Biochemistry and Physiology Part A: Molecular & Integrative Physiology* 192 (February 2016): 52–56. <https://doi.org/10.1016/j.cbpa.2015.11.015>.

Costanzo, Jon P. ‘A Physiological Basis for Prolonged Submergence in Hibernating Garter Snakes *Thamnophis Sirtalis:* Evidence for an Energy-Sparing Adaptation’. *Physiological Zoology* 62, no. 2 (March 1989): 580–92. <https://doi.org/10.1086/physzool.62.2.30156186>.

DePari, J.A. ‘Overwintering in the Nest Chamber by Hatcling Painted Turtles, *Chrysemys Picta*, in Northern New Jersey’. *Chelonian Conservation and Biology* 25 (1996): 12.

Lutterschmidt, Deborah I., Ashley R. Lucas, and Andrew R. Summers. ‘Trans‐seasonal Activation of the Neuroendocrine Reproductive Axis: Low‐temperature Winter Dormancy Modulates Gonadotropin‐releasing Hormone Neurons in Garter Snakes’. *Journal of Experimental Zoology Part A: Ecological and Integrative Physiology* 337, no. 1 (January 2022): 50–64. <https://doi.org/10.1002/jez.2506>.

Lutterschmidt, D. I., and R. T. Mason. ‘Endocrine Mechanisms Mediating Temperature-Induced Reproductive Behavior in Red-Sided Garter Snakes (*Thamnophis Sirtalis Parietalis*)’. *Journal of Experimental Biology* 212, no. 19 (1 October 2009): 3108–18. <https://doi.org/10.1242/jeb.033100>.

Mitchell, T.S., J.M. Refsnider, A. Sethuraman, D.A. Warner, and F.J. Janzen. ‘Experimental Assessment of Winter Conditions on Turtle Nesting Behaviour’. *Evolutionary Ecology Research* 18 (2017): 271–80.

Muir, Timothy J., Brian D. Dishong, Richard E. Lee, and Jon P. Costanzo. ‘Energy Use and Management of Energy Reserves in Hatchling Turtles (*Chrysemys picta*) Exposed to Variable Winter Conditions’. *Journal of Thermal Biology* 38, no. 6 (August 2013): 324–30. <https://doi.org/10.1016/j.jtherbio.2013.04.003>.

Ruby, D.E. ‘Winter Activity in Yarrow’s Spiny Lizard, *Sceloporus jarrovi’*. *Herpetologica* 33, no. 3 (1977): 322–33.

Spencer, R.-J., and F. J. Janzen. ‘A Novel Hypothesis for the Adaptive Maintenance of Environmental Sex Determination in a Turtle’. *Proceedings of the Royal Society B: Biological Sciences* 281, no. 1789 (22 August 2014): 20140831. <https://doi.org/10.1098/rspb.2014.0831>.

Zani, Peter A. ‘Climate Change Trade‐Offs in the Side‐Blotched Lizard (*Uta Stansburiana* ): Effects of Growing‐Season Length and Mild Temperatures on Winter Survival’. *Physiological and Biochemical Zoology* 81, no. 6 (November 2008): 797–809. <https://doi.org/10.1086/588305>.

Zani, P. A., J. T. Irwin, M. E. Rollyson, J. L. Counihan, S. D. Healas, E. K. Lloyd, L. C. Kojanis, B. Fried, and J. Sherma. ‘Glycogen, Not Dehydration or Lipids, Limits Winter Survival of Side-Blotched Lizards *(Uta stansburiana*)’. *Journal of Experimental Biology* 215, no. 17 (1 September 2012): 3126–34. <https://doi.org/10.1242/jeb.069617>.

b) Observational dataset

Altwegg, Res, Stefan Dummermuth, Bradley R. Anholt, and Thomas Flatt. ‘Winter Weather Affects Asp Viper *Vipera aspis* Population Dynamics through Susceptible Juveniles’. *Oikos* 110, no. 1 (July 2005): 55–66. <https://doi.org/10.1111/j.0030-1299.2001.13723.x>.

Axelsson, Jannike, Erik Wapstra, Emily Miller, Nicky Rollings, and Mats Olsson. ‘Contrasting Seasonal Patterns of Telomere Dynamics in Response to Environmental Conditions in the Ectothermic Sand Lizard, *Lacerta agilis’*. *Scientific Reports* 10, no. 1 (December 2020): 182. <https://doi.org/10.1038/s41598-019-57084-5>.

Baker, Patrick Joseph, John B. Iverson, Richard E. Lee, and Jon P. Costanzo. ‘Winter Severity and Phenology of Spring Emergence from the Nest in Freshwater Turtles’. *Naturwissenschaften* 97, no. 7 (July 2010): 607–15. <https://doi.org/10.1007/s00114-010-0675-x>.

Bull, C. Michael, and Dale Burzacott. ‘Changes in Climate and in the Timing of Pairing of the Australian Lizard, *Tiliqua Rugosa* : A 15‐year Study’. *Journal of Zoology* 256, no. 3 (March 2002): 383–87. <https://doi.org/10.1017/S0952836902000420>.

Costanzo, Jon P., Stephen A. Dinkelacker, John B. Iverson, and Richard E. Lee, Jr. ‘Physiological Ecology of Overwintering in the Hatchling Painted Turtle: Multiple‐Scale Variation in Response to Environmental Stress’. *Physiological and Biochemical Zoology* 77, no. 1 (January 2004): 74–99. <https://doi.org/10.1086/378141>.

Janzen, Fredric J., Luke A. Hoekstra, Ronald J. Brooks, David M. Carroll, J. Whitfield Gibbons, Judith L. Greene, John B. Iverson, et al. ‘Altered Spring Phenology of North American Freshwater Turtles and the Importance of Representative Populations’. *Ecology and Evolution* 8, no. 11 (June 2018): 5815–27. <https://doi.org/10.1002/ece3.4120>.

Jones, Alice R., C. Michael Bull, Barry W. Brook, Konstans Wells, Kenneth H. Pollock, and Damien A. Fordham. ‘Tick Exposure and Extreme Climate Events Impact Survival and Threaten the Persistence of a Long-Lived Lizard’. Edited by Bethany Hoye. *Journal of Animal Ecology* 85, no. 2 (March 2016): 598–610. <https://doi.org/10.1111/1365-2656.12469>.

Kissner, Kelley J., and Patrick J. Weatherhead. ‘Phenotypic Effects on Survival of Neonatal Northern Watersnakes *Nerodia sipedon’*. *Journal of Animal Ecology* 74, no. 2 (2005): 259–65.

McGaugh, S.E. ‘Micro-Evolutionary Potential of Temperature-Dependent Sex Determination in a Wild Population of Painted Turtles, *Chrysemys picta’*. PhD, Iowa State University, 2009.

Milanovich, Joseph R., Brock P. Struecker, Stanislaw A. Warcholek, and Leigh Anne Harden. ‘Thermal Environment and Microhabitat of Ornate Box Turtle Hibernacula’. *Wildlife Biology* 2017, no. 1 (January 2017): wlb.00295. <https://doi.org/10.2981/wlb.00295>.

Mitchell, T.S., J.M. Refsnider, A. Sethuraman, D.A. Warner, and F.J. Janzen. ‘Experimental Assessment of Winter Conditions on Turtle Nesting Behaviour’. *Evolutionary Ecology Research* 18 (2017): 271–80.

Nagle, R.D., O.M. Kinney, J.D. Congdon, and C.W. Beck. ‘Winter Survivorship of Hatchling Painted Turtles (*Chrysemys Picta* ) in Michigan’. *Canadian Journal of Zoology* 78, no. 2 (5 March 2000): 226–33. <https://doi.org/10.1139/z99-206>.

Nordberg, E.J., and V.A. Cobb. ‘Body Temperatures and Winter Activity in Overwintering Timber Rattlesnakes (*Crotalus horridus*) in Tennessee, USA’. *Herpetological Conservation and Biology* 12, no. 3 (2017): 606–15.

Packard, Gary C. ‘Temperatures during Winter in Nests with Hatchling Painted Turtles (*Chrysemys picta*)’. *Herpetologica* 53, no. 1 (1997): 89–95.

Prodon, Roger, Philippe Geniez, Marc Cheylan, and Aurélien Besnard. ‘Amphibian and Reptile Phenology: The End of the Warming Hiatus and the Influence of the NAO in the North Mediterranean’. *International Journal of Biometeorology* 64, no. 3 (March 2020): 423–32. <https://doi.org/10.1007/s00484-019-01827-6>.

Rugiero, Lorenzo, Giuliano Milana, Fabio Petrozzi, Massimo Capula, and Luca Luiselli. ‘Climate-Change-Related Shifts in Annual Phenology of a Temperate Snake during the Last 20 Years’. *Acta Oecologica* 51 (August 2013): 42–48. <https://doi.org/10.1016/j.actao.2013.05.005>.

Schuett, Gordon W., Roger A. Repp, Emily N. Taylor, Dale F. DeNardo, Ryan L. Earley, Edward A. Van Kirk, and William J. Murdoch. ‘Winter Profile of Plasma Sex Steroid Levels in Free-Living Male Western Diamond-Backed Rattlesnakes, *Crotalus atrox* (Serpentes: Viperidae)’. *General and Comparative Endocrinology* 149, no. 1 (October 2006): 72–80. <https://doi.org/10.1016/j.ygcen.2006.05.005>.

Schwanz, Lisa E., and Fredric J. Janzen. ‘Climate Change and Temperature‐Dependent Sex Determination: Can Individual Plasticity in Nesting Phenology Prevent Extreme Sex Ratios?’ *Physiological and Biochemical Zoology* 81, no. 6 (November 2008): 826–34. <https://doi.org/10.1086/590220>.

Sperry, J. H., and P. J. Weatherhead. ‘Individual and Sex-Based Differences in Behaviour and Ecology of Rat Snakes in Winter: Rat Snake Winter Ecology’. Edited by Robert Knell. *Journal of Zoology* 287, no. 2 (June 2012): 142–49. <https://doi.org/10.1111/j.1469-7998.2011.00895.x>.

Turner, Rebecca K., and Ilya M. D. Maclean. ‘Microclimate‐driven Trends in Spring‐emergence Phenology in a Temperate Reptile (*Vipera berus*): Evidence for a Potential “Climate Trap”?’ *Ecology and Evolution* 12, no. 2 (February 2022). <https://doi.org/10.1002/ece3.8623>.

Vicente Liz, A., V. Santos, T. Ribeiro, M. Guimarães, and L. Verrastro. ‘Are Lizards Sensitive to Anomalous Seasonal Temperatures? Long-Term Thermobiological Variability in a Subtropical Species’. Edited by William David Halliday. *PLOS ONE* 14, no. 12 (19 December 2019): e0226399. <https://doi.org/10.1371/journal.pone.0226399>.

Willette, Demian A.S, John K Tucker, and Fredric J Janzen. ‘Linking Climate and Physiology at the Population Level for a Key Life-History Stage of Turtles’. *Canadian Journal of Zoology* 83, no. 6 (1 June 2005): 845–50. <https://doi.org/10.1139/z05-078>.
